# Integration of molecular docking, molecular dynamics and network pharmacology to explore the multi‐target pharmacology of fenugreek against diabetes

**DOI:** 10.1111/jcmm.17787

**Published:** 2023-05-31

**Authors:** Wenfeng Luo, Jie Deng, Jiecheng He, Liang Yin, Rong You, Lingkun Zhang, Jian Shen, Zeping Han, Fangmei Xie, Jinhua He, Yanqing Guan

**Affiliations:** ^1^ School of Life Science South China Normal University Guangzhou China; ^2^ South China Normal University‐Panyu Central Hospital Joint Laboratory of Translational Medical Research Panyu Central Hospital Guangzhou China; ^3^ Medical Imaging Institute of Panyu Guangzhou China; ^4^ Shunde Polytecnic Foshan China

**Keywords:** anti‐inflammatory, anti‐oxidative stress, diabetes, fenugreek, molecular docking, network pharmacology

## Abstract

Fenugreek is an ancient herb that has been used for centuries to treat diabetes. However, how the fenugreek‐derived chemical compounds work in treating diabetes remains unclarified. Herein, we integrate molecular docking and network pharmacology to elucidate the active constituents and potential mechanisms of fenugreek against diabetes. First, 19 active compounds from fenugreek and 71 key diabetes‐related targets were identified through network pharmacology analysis. Then, molecular docking and simulations results suggest diosgenin, luteolin and quercetin against diabetes via regulation of the genes ESR1, CAV1, VEGFA, TP53, CAT, AKT1, IL6 and IL1. These compounds and genes may be key factors of fenugreek in treating diabetes. Cells results demonstrate that fenugreek has good biological safety and can effectively improve the glucose consumption of IR‐HepG2 cells. Pathway enrichment analysis revealed that the anti‐diabetic effect of fenugreek was regulated by the AGE‐RAGE and NF‐κB signalling pathways. It is mainly associated with anti‐oxidative stress, anti‐inflammatory response and β‐cell protection. Our study identified the active constituents and potential signalling pathways involved in the anti‐diabetic effect of fenugreek. These findings provide a theoretical basis for understanding the mechanism of the anti‐diabetic effect of fenugreek. Finally, this study may help for developing anti‐diabetic dietary supplements or drugs based on fenugreek.

## 1 INTRODUCTION

Diabetes is the third biggest health threat after cardiovascular disease and cancer, according to the International Diabetes Federation.^1^, ^2^ Statistics from the World Health Organization indicate that 415 million people had diabetes in 2015, and the prevalence of diabetes is predicted to rise to 693 million by 2045.^2^ Depending on the pathogenesis, diabetes can be broadly divided into Type 1 diabetes mellitus (T1DM), T2DM, gestational diabetes and other specific types; of these, T2DM accounts for more than 90% of cases.^3^ T2DM is a chronic disease in which the negative feedback cycle between insulin activity and insulin secretion becomes dysregulated, resulting in abnormal glucose metabolism.^4^ In addition, the long‐term accumulated effects of abnormal glucose metabolism lead to cardiovascular injury, kidney disease, diabetic foot and other complications, which can have serious impacts on the mental and physical well‐being of T2DM patients.^5^ Lifelong medication is usually necessary for people with diabetes to control their blood glucose, and thus their health care costs are three times more than that of people without diabetes. In 2015, 67.3 billion USD was spent worldwide to treat diabetes, accounting for 12% of global health spending.^6^, ^7^ Therefore, there is an urgent need to develop effective ways to prevent and treat diabetes.

Fenugreek (*Trigonella foenum‐graecum* L.) is a plant in the Luminaceae family and is native to India and North Africa.^8^ It is cultivated in many parts of the world, including China, Australia, northern Africa, Europe and Argentina.^9^ Fenugreek has been used as both a food and medicine in Iran, India and China for about six millennia.^10^ Pharmacological studies have revealed that fenugreek contains alkaloids, flavonoids, polysaccharides, steroidal saponins and volatile oils, as well as other active constituents with lipid‐lowering and hypoglycaemic effects.^11^, ^12^, ^13^ Fenugreek is widely used as a natural dietary supplement for the treatment of diabetes,^14^ for which it has been shown to be safe and effective.^15^ Many studies have demonstrated that fenugreek‐derived alkaloids not only show strong β‐glucosidase inhibition, cancer suppression, antioxidant and anti‐inflammatory activities but also exert effects such as cardiovascular protection, cholesterol reduction and adipogenesis inhibition.^16^ Moreover, the flavonoids, polysaccharides and saponins derived from fenugreek have been shown to play a role in controlling blood glucose levels as well as treating diabetes and its complications in both patients and animal models.^17^ However, the exact mechanism remains unclear.

Details on the reactions between specific active constituents in fenugreek and cells, genes and proteins in the body remain limited. Therefore, an association analysis of the physicochemical properties of fenugreek along with potential targets and their possible mechanisms is needed. Recently, network pharmacology has emerged as an exciting new subfield of drug development because it combines analysis of biomedical big data with systems medicine.^18^, ^19^ By introducing biomedical big data, the network relationships between active compounds in traditional drugs and their target proteins can be constructed, thereby revealing the mechanism of synergistic therapeutic action in traditional medicines. Thus, network pharmacology has helped to transition the drug discovery field from the conventional ‘one target, one drug’ framework to a ‘network target, multi‐compound treatment’ approach.^20^, ^21^

Fenugreek has long been used as a traditional Chinese herbal medicine for the treatment of diabetes. Recently, Oh et al.^22^ investigated the anti‐diabetic effect of garlic shell based on molecular docking and network pharmacology and reported the bioactive constituents and signalling pathways against diabetes, demonstrating the usefulness of these techniques in identifying the bioactive constituents of traditional herbal formulas, the potential targets of action and the mechanisms of interaction between them.

**FIGURE 1:**
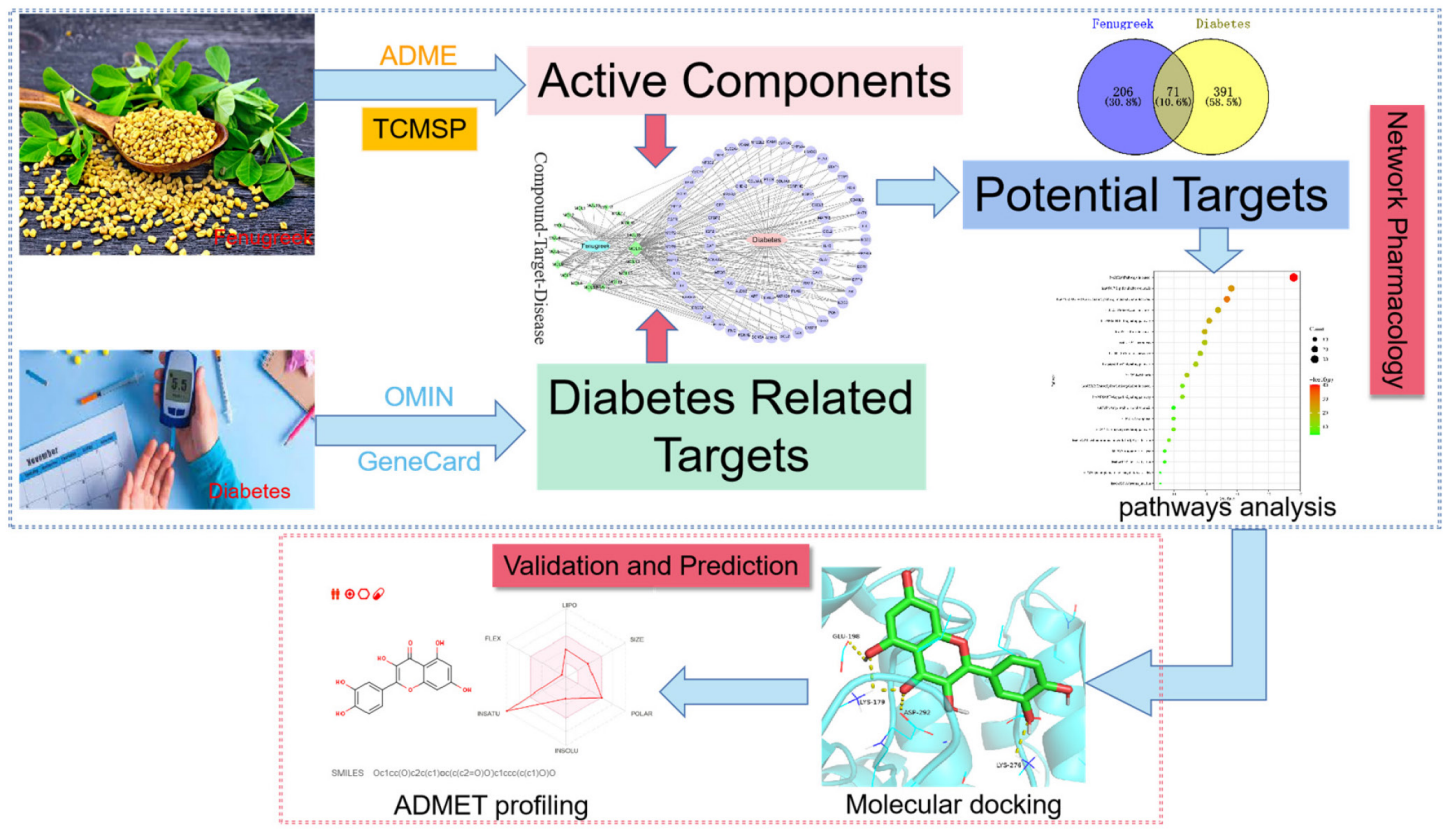
Study workflow comprising a network pharmacology stage and a validation and prediction stage, aimed at elucidating the mechanisms underlying the anti‐diabetic effects of fenugreek.

In the present study, we used molecular docking, molecular dynamics (MD) simulation and network pharmacology to investigate the active constituents of fenugreek and identify their potential target proteins, with the aim of elucidating the underlying mechanisms of its anti‐diabetic activities. The study workflow is shown in Figure 1.

## 2 MATERIALS AND METHODS

### 2.1 Screening of active compounds and related targets

A list of compounds in fenugreek was obtained from the Traditional Chinese Medicine Systems Pharmacology (TCMSP) database (http://lsp.nwu.edu.cn/tcmsp.php/).^23^ Screening of active constituents was performed following the principle that drug‐likeness (DL) is ≥0.18 and oral bioavailability is ≥30%. Compounds that were not identified by screening but that have been reported as metabolic regulators were also included in the network analysis. Then, the TCMSP database was searched with the aim of further predicting the targets of the active constituents. To generate a more comprehensive collection, the simplified molecular‐input line‐entry system (SMILES) entry for each active constituents was retrieved from the PubChem database. Next, the BindingDB (http://bindingdb. org/bind/index.jsp), DrugBank (https://go.drugbank.com/), STITCH (http://stitch.embl.de/) and SwissTargetPrediction (http://www.swisstargetprediction.ch/) databases were searched for related targets of active compounds based on their SMILES formulas.

### 2.2 Predicting targets of diabetes

Searches were conducted of GeneCards (https: //www.genecards. org/) and OMIM (https:// www.omim.org/), using the keywords ‘diabetes’ and ‘hyperlipidemia,’ respectively, with the aim of obtaining the related targets of diseases in *Homo sapiens*. Of the targets obtained from GeneCards, those with a relevance score > 40 were selected as potential targets. As for the OMIM datasets, all of the targets related to diabetes were involved. Next, an online Venn diagram drawing tool, Venny ver. 2.1 (https://bioinfogp.cnb.csic.es/tools/venny/), was used to visualize the overlapping targets between the selected compounds and diabetes target genes.

### 2.3 Network construction

#### 2.3.1 Drug‐Compound‐Target‐Disease network

The overlapping targets and active compounds were imported into Cytoscape ver. 3.7.1 in order to construct the Drug‐Compound‐Target‐Disease network and the ‘Network Analyzer’ function was used to analyse the topological properties of the network.

#### 2.3.2 Protein‐Protein Interaction (PPI) network

Protein‐protein interactions were analysed using String ver. 11.0 (https://string‐db.org/). To further investigate the mechanism of action of fenugreek in the treatment of T2DM, the intersection targets were imported into String. The minimum combined score was set to 0.400 and protein‐interaction relationships for *Homo sapiens* were obtained. Targets without interaction were removed and the data were saved as SIF files. Next, the PPI network was constructed by importing node1, node2 and the combined score into Cytoscape. Finally, the core targets were screened by cluster analysis using the MCODE plug‐in in Cytoscape.

#### 2.3.3 GO and KEGG pathways enrichment analyses

Gene Ontology (GO) enrichment analysis was performed by running the ‘cluster Profiler’ package in R ver. (R Foundation for Statistical Computing). The Biological Process (BP), Cellular Component (CC) and Molecular Function (MF) modules were selected and significant biological annotations (*p* < 0.05) were chosen for analysis. At the same time, KEGG pathway enrichment analysis was performed to investigate the major signalling pathways involved in the anti‐diabetic effects of fenugreek. Finally, the enriched results were analysed using the ‘ggplot2’ package in R.

#### 2.3.4 Compound‐Gene‐Pathway network

To clarify the relationship among active compounds, target genes and pathways, the top 20 significant pathways were intersected with the Compound‐Gene‐Disease network to construct the Compound‐Gene‐Pathway network. The results were visualized in Cytoscape.

### 2.4 Molecular docking and ADMET profiling

Molecular docking can help to validate key targets in network pharmacology.^24^ First, we searched the important compounds in the PubChem database, and the structure of ‘MOL2’ was exported. Next, the 3D structures of the core proteins were obtained from the Protein Data Bank (https://www.rcsb.org/). Then, the progress of dehydration, hydrogenation and charge editing were monitored using AutoDock software 4.2.6, and the modified target proteins and important compounds were exported as PDBQT files. Next, docking progress was monitored separately using AutoDock Vina software 1.2.0. Finally, the docking results were imported into PyMol software for analysis and visualization. Here, we employed Metformin, a commonly used treatment drug for diabetes mellitus, as reference drug.

The SwissADME online server was used to perform machine learning to predict the drug‐like properties. The six important compounds were input into the SwissADME website and the ADMET properties were selected for analysis. The toxicity of the six important compounds was predicted using the free online tool ProTox‐II, and the results, including predicted LD_50_, reverse mutation assay (Ames test) and cytotoxicity, were recorded.

### 2.5 Molecular dynamics simulations

GROMACS (2021.3) with CHARMm36 force field were employed for MD simulations. According to docking results, the top three protein‐ligand complexes in each group were selected for MD simulations. Protein‐inhibitor complex from PDB datebase were employed as the reference systems. Simulate parameters were taken as described.^25^, ^26^ A total of 10 ns MD simulation was conducted. The data for every one picosecond was saved. Xmgrace were utilized to analyse RMSD and RMSF of each complex. GMX‐MM/PBSA (Version 1.6.0) was recruited to compute the binding free energy calculations.^27^

### 2.6 In vitro validation

HepG2 cells were used to establish insulin resistance model. As the method described by Chen et al.^28^ Briefly, HepG2 cells were planted into 96‐well plates with a density of 1 × 10^5^ cells/well and cultured in the cell incubator for 24 h. Then, cells were starved for 24 h by serum‐free medium. The serum‐free medium was then replaced with high‐glucose DMEM (50 μM) with recombinant human insulin (5 × 10^−7^ M) for 48 h to establish IR model.

100 μL of HepG2 cells (1 × 10^6^ cells/ml) were added into 96‐well plates. After 24 h pre‐adhesion, the original medium was replaced with fenugreek solution at different concentrations (12.5, 25, 50, 100, 200, 400, 800 and 1600 μg/mL). Cells were cultured for another 24 h and 48 h. Then, CCK‐8 test were performed to evaluate the cell viability.

As mentioned above, IR‐HepG2 model were induced by high glucose DMEM with recombinant human insulin. Then, the IR‐HepG2 cells were cultured with different concentrations of fenugreek for 24 h. Subsequently, glucose uptake assays were carried out using a glucose assay kit (glucose oxidase method) to estimate the anti‐diabetic effects of fenugreek.

## 3 RESULTS

### 3.1 Active constituents in fenugreek

Searching the TCMSP database, 14 putative compounds derived from fenugreek, with a threshold value OB ≥30% and DL ≥0.18, were identified by screening. Meanwhile a search of the literature was conducted, and five active constituents were identified. The basic characteristics of all 19 active constituents are presented in Table 1. These 19 active constituents are mainly terpenoids, flavonoids and organic acids.

**TABLE 1:** Characteristics of 19 active constituents derived from fenugreek.

MOL‐ID	PubChem ID	Molecular name	Mw	OB (%)	DL	AlogP	Type
MOL1	64,971	Mairin	456.78	55.38	0.78	6.52	Terpenoid
MOL2	222,284	β‐sitosterol	414.79	36.91	0.75	8.08	Terpenoid
MOL3	5,281,779	Irolone	298.26	46.87	0.36	2.1	Flavonoid
MOL4	5,280,378	Formononetin	268.28	69.67	0.21	2.58	Flavonoid
MOL5	5,280,448	Calycosin	284.28	47.75	0.24	2.32	Flavonoid
MOL6	5,280,863	Kaempferol	286.25	41.88	0.24	1.77	Flavonoid
MOL7	99,474	Diosgenin	414.69	80.88	0.81	4.63	Terpenoid
MOL8	162,995,045	Gallic acid‐3‐O‐(6’‐O‐galloyl)‐glucoside	484.4	30.25	0.67	−0.03	Glycoside
MOL9	5,280,445	Luteolin	286.25	36.16	0.25	2.07	Flavonoid
MOL10	5,281,471	Hiyodori lactone A	420.5	58.33	0.52	1.81	Flavonoid
MOL11	99,516	Tigogenone	414.69	37.35	0.81	4.56	Terpenoid
MOL12	162,350	Isovitexin	432.41	31.29	0.72	−0.06	Flavonoid
MOL13	12,304,433	Neotigogenin	416.71	80.98	0.81	4.88	Terpenoid
MOL14	5,280,343	Quercetin	302.25	46.43	0.28	1.5	Flavonoid
MOL15	442,664	Vicenin‐2	594.57	3.42	0.78	−2.45	Flavonoid
MOL16	442,664	Vitexin‐7‐glucoside_qt	432.41	16.49	0.71	−0.06	Flavonoid
MOL17	114,776	Isoorientin	448.41	23.30	0.76	−0.32	Flavonoid
MOL18	5,280,441	Vitexin	432.41	3.05	0.71	−0.06	Flavonoid
MOL19	2,773,624	4‐Hydroxyisoleucine	147.17	/	/	−2.50	Amino Acid

Abbreviations: AlogP, atomic partition coefficient; DL, drug‐likeness; OB, oral bioavailability; Mw, molecular weight.

### 3.2 Intersection of fenugreek and diabetes targets

**FIGURE 2:**
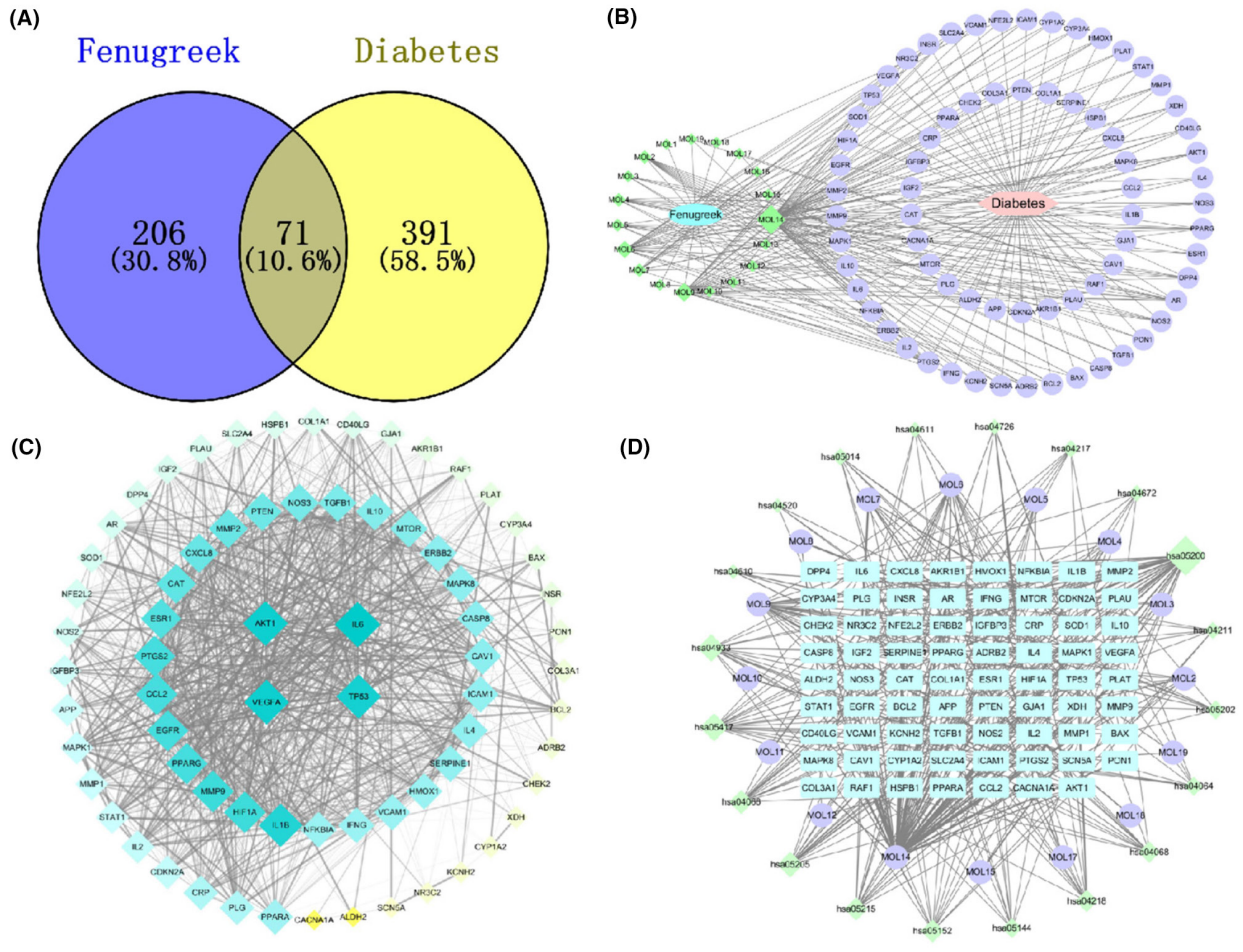
Network pharmacology analyse results. (A) Venn diagram of fenugreek–diabetes targets. (B) Drug‐Compound‐Target‐Disease interaction network. Green diamonds are active compounds, the pink hexagon is the disease, purple circles are the common target genes of the compounds and the disease and the lines represent interactions between compounds and targets. (C) PPI network of the common target genes. Diamonds represent proteins, the colours (from blue to green to yellow) indicate the degree of binding between proteins and the lines represent protein‐protein interactions. (D) Compound‐Gene‐Pathway network. Blue rectangles represent hub genes, purple circles represent active compounds and green diamonds indicate pathways associated with the core targets.

After exploring the TCMSP, SwissTargetPrediction, DrugBank and other databases. 277 diabetes‐related targets without duplicate values were obtained. Meanwhile, a total of 462 diabetes targets were identified in the GeneCards and OMIM databases, using diabetes‐related target screening rules. As shown in Figure 2A, a total of 71 overlapping targets were obtained by determining the intersection of the 277 compound‐related targets and the 462 diabetes‐related targets by using Venny.

### 3.3 Construction and analysis of the Drug–Compound–Target–Disease network

To construct the Drug–Compound–Target–Disease network (Figure 2B), 71 common targets of fenugreek in diabetes treatment were input to Cytoscape. As shown in Figure 2B, the network comprised 98 nodes (19 active compound nodes, 71 target nodes, 1 fenugreek node and 1 diabetes node) and 252 edges, each representing the relationship between fenugreek and the active compounds, the active compounds and the targets and the target and the disease (i.e., diabetes). In the network, the degree value represents the number of edges connected to the node. Compound nodes are screened according to the principle of network topology. These compounds play a key role in the whole network^13^ and may be the key compounds in drugs for treating diabetes. The median value of the compound degree in this network was four, and the important compounds were MOL7, MOL4, MOL2, MOL6, MOL9 and MOL14, with 10, 10, 10, 22, 27 and 60 targets (Table 2), respectively. The degree distribution and the centrality distribution of the important compound nodes in the network indicate importance in the treatment of diabetes.

**TABLE 2:** Topology parameters of important compounds in the network.

MOL ID	Chemical name	Degree	Betweenness centrality
MOL7	Diosgenin	10	0.01457813
MOL4	Formononetin	10	0.00951153
MOL2	β‐Sitosterol	10	0.01139956
MOL6	Kaempferol	22	0.03922565
MOL9	Luteolin	27	0.05734171
MOL14	Quercetin	60	0.30288612

### 3.4 PPI network of therapeutic targets for fenugreek against diabetes

A total of 71 common targets were input to the String database, and the PPI network was exported as a TSV file to Cytoscape, where the PPI network was visualized and analysed. As shown in Figure 2C, the PPI network comprised 71 nodes and 2252 edges, with the nodes indicating potential targets and edges representing the relation between the two connected targets. In addition, the size and colour were proportional to the degree value of the nodes, with blue indicating larger values and yellow indicating smaller values. We found that the median degree value was 33. Based on a previous study, we set the screening conditions as degree >40 and betweenness centrality >0.024. The eight key target genes obtained, ranked according to decreasing node degree, were IL6 (61), AKT1 (59), VEGFA (59), TP53 (58), IL1B (56), ESR1 (49), CAT (47) and CAV1 (41). The characteristics of these key genes are presented in Table 3.

**TABLE 3:** Characteristics of key genes.

Target name	Gene symbol	Degree	Betweenness centrality
ESR1	Oestrogen receptor 1	49	0.02681766
CAV1	Caveolin 1	41	0.06836266
VEGFA	Vascular endothelial growth factor A	59	0.0283019
TP53	Tumour protein P53	58	0.02667203
CAT	Catalase	47	0.03529778
AKT1	AKT Serine/threonine kinase 1	59	0.02828517
IL6	Interleukin 6	61	0.04171243
IL1B	Interleukin 1 beta	56	0.02426754

### 3.5 GO and KEGG pathway enrichment analysis

**FIGURE 3:**
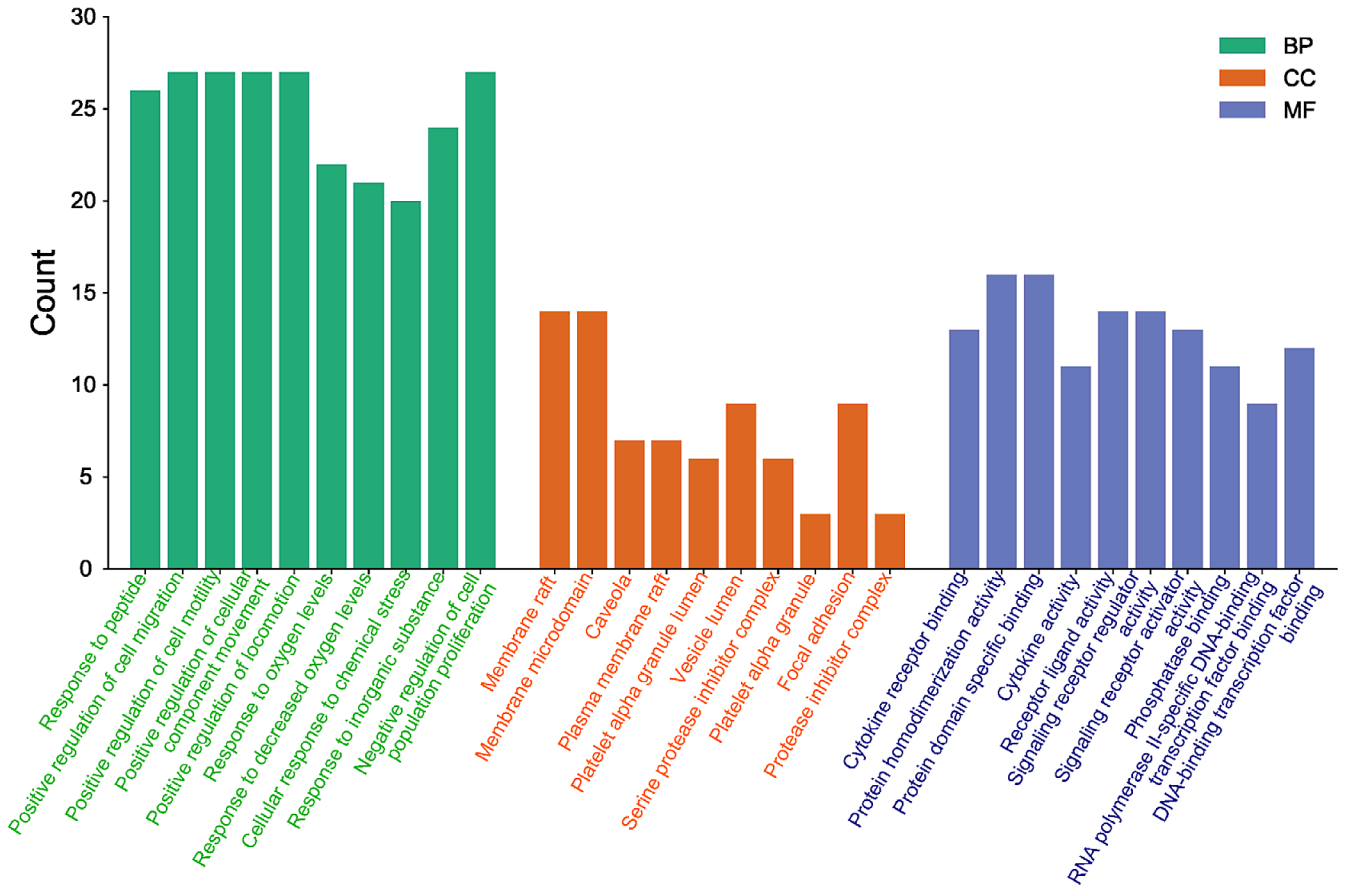
Visualization of GO term enrichment.

GO functional enrichment analysis was carried out using the Database for Annotation, Visualization and Integrated Discovery. A total of 1713 GO terms (*p* < 0.01) were enriched, including 1553 for BP, 61 for CC and 99 for MF. Top 10 GO terms ranked by *p* value for BP, CC and MF were plotted in a graph, for a total of 30. In Figure 3, the ordinate indicates the gene number of enriched GO terms. The top‐ranked GO terms were response to peptide, positive regulation of cell migration, response to oxygen levels, cellular response to chemical stress, negative regulation of cell population proliferation, regulation of cell adhesion, gland development, positive regulation of protein phosphorylation, regulation of apoptotic signalling pathway and circulatory system process.

**FIGURE 4:**
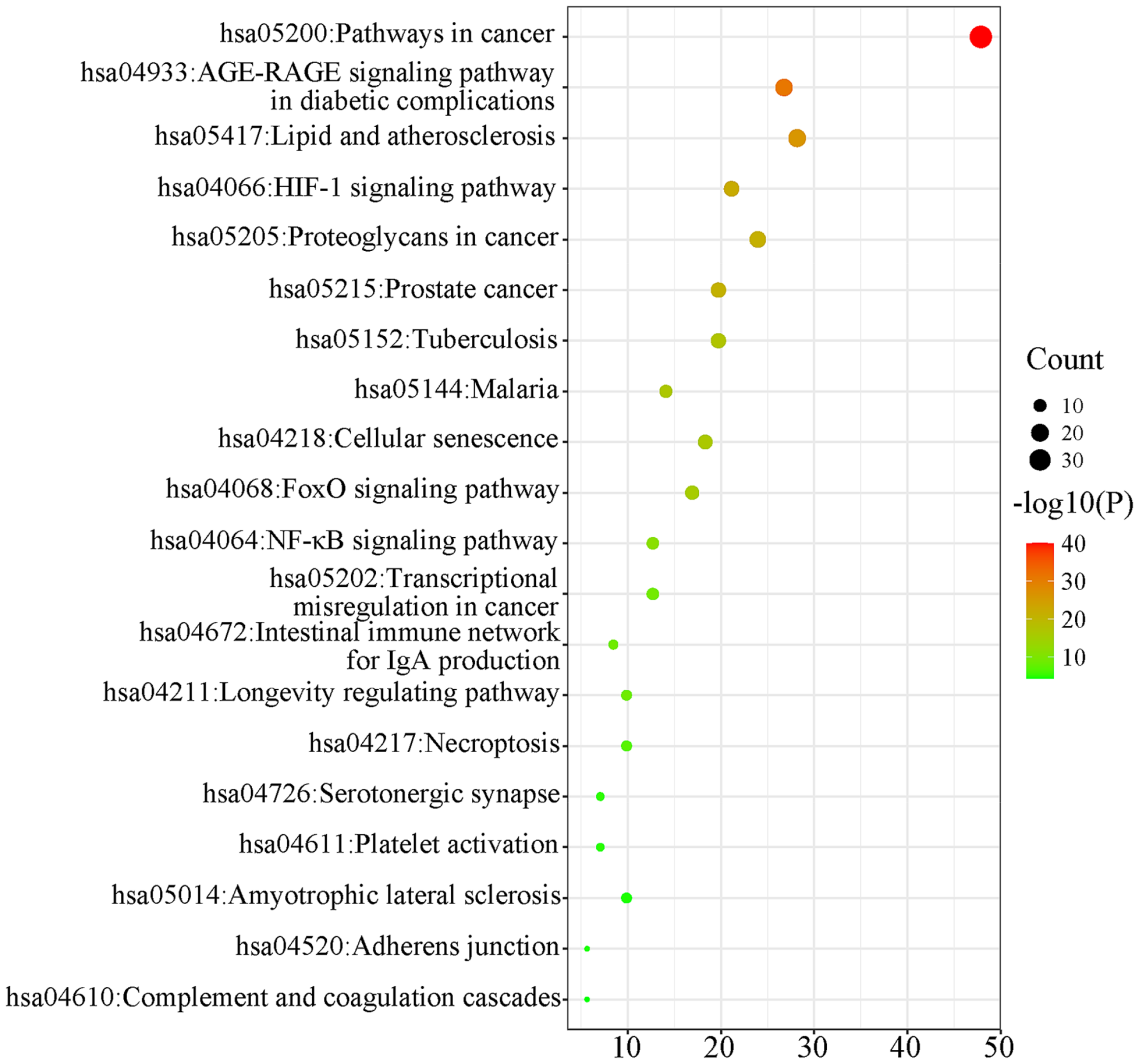
Visualization of KEGG pathway enrichment.

A total of 168 KEGG pathways were enriched by inputting 71 core genes to the ‘clusterProfiler’ package in R. The top 20 pathways ranked according to *p* value are presented in Table 4, and the top 20 pathways are visualized in Figure 4. The size of the circle indicates the number of correlated genes in the pathway, and the colour indicates the *p*‐value, with green indicating smaller values and red indicating larger values. The network includes mainly cancer pathways, the AGE‐RAGE signalling pathway, the FoxO signalling pathway, the HIF‐1 signalling pathway, the NF‐κB signalling pathway and lipids and atherosclerosis, which suggests that fenugreek might exert its anti‐diabetic effects through these pathways.

**TABLE 4:** Top 20 KEGG pathways ranked by *p* value.

Pathway	Gene ratio (%)	Count	Log10(P)	Log10(q)
hsa05200:Pathways in cancer	47.89	34	−40.07	−37.53
hsa04933:AGE‐RAGE signalling pathway in diabetic complications	26.76	19	−31.03	−28.79
hsa05417:Lipid and atherosclerosis	28.17	20	−26.13	−24.07
hsa04066:HIF‐1 signalling pathway	21.13	15	−22.18	−20.42
hsa05205:Proteoglycans in cancer	23.94	17	−21.28	−19.58
hsa05215:Prostate cancer	19.72	14	−21.01	−19.43
hsa05152:Tuberculosis	19.72	14	−17.12	−15.86
hsa05144:Malaria	14.08	10	−16.6	−15.4
hsa04218:Cellular senescence	18.31	13	−16.3	−15.17
hsa04068:FoxO signalling pathway	16.9	12	−15.56	−14.5
hsa04064:NF‐kappa B signalling pathway	12.68	9	−11.52	−10.74
hsa05202:Transcriptional misregulation in cancer	12.68	9	−9.11	−8.47
hsa04211:Longevity regulating pathway	9.86	7	−8.76	−8.16
hsa04672:Intestinal immune network for IgA production	8.45	6	−8.76	−8.16
hsa04217:Necroptosis	9.86	7	−7.01	−6.47
hsa04726:Serotonergic synapse	7.04	5	−5.11	−4.64
hsa04611:Platelet activation	7.04	5	−4.95	−4.5
hsa05014:Amyotrophic lateral sclerosis	9.86	7	−4.62	−4.19
hsa04520:Adherens junction	5.63	4	−4.62	−4.19
hsa04610:Complement and coagulation cascades	5.63	4	−4.31	−3.9

### 3.6 Compound‐Gene‐Pathway network analysis

**FIGURE 5:**
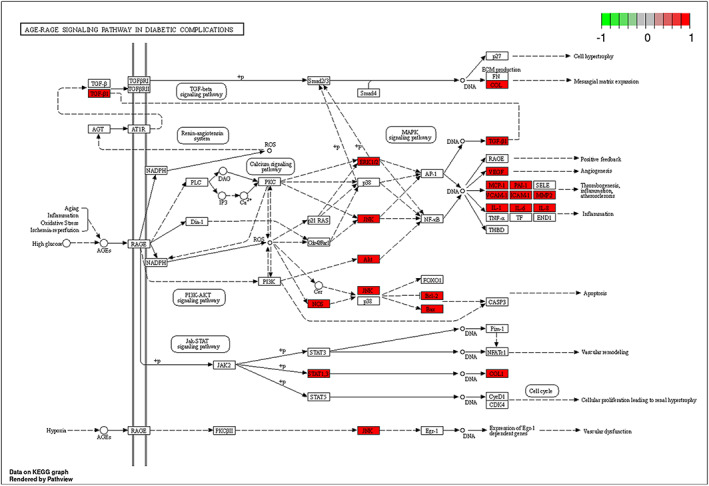
Action of the core target on the AGE‐RAGE signalling pathway in diabetic complications. Red rectangles represent the key targets.

The Compound‐Gene‐Pathway network was constructed using Cytoscape. As shown in Figure 2D, the node size represents the degree value. The network comprises 110 nodes and 390 edges. AKT1, PTGS2, MAPK1, TP53 and IL‐6 interacted with most of the potential pathways, including cancer pathways, the AGE‐RAGE signalling pathway, the FoxO signalling pathway, the HIF‐1 signalling pathway, the NF‐κB signalling pathway and lipids and atherosclerosis, which suggests that these genes might play a key role in the anti‐diabetic effects of fenugreek. Figure 5 shows the action of the core target on the AGE‐RAGE signalling pathway in diabetic complications.

### 3.7 Molecular docking verification and ADMET profiling

**FIGURE 6:**
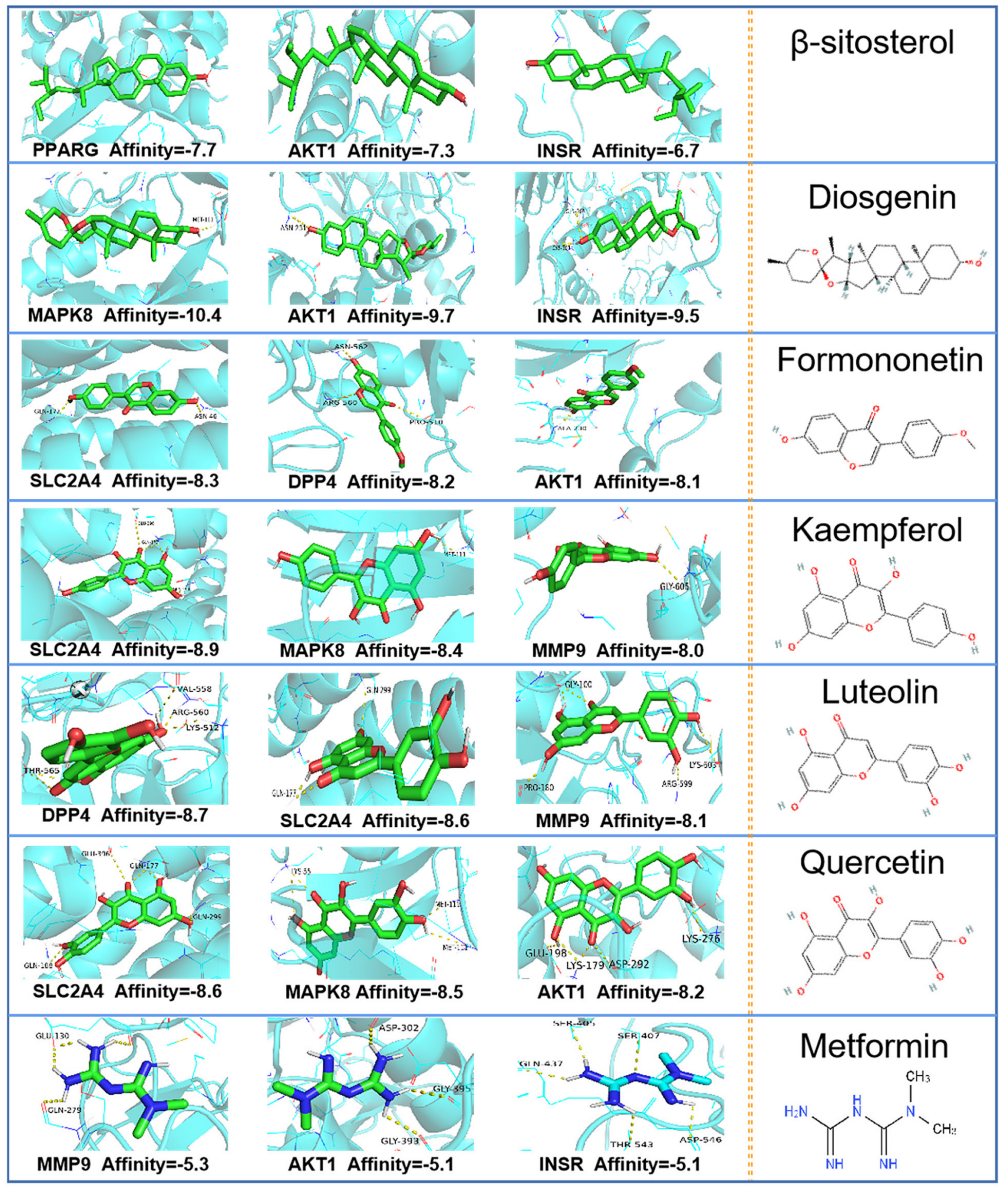
Molecular docking results of the important compounds and the corresponding proteins of the gene targets.

The six important active constituents—β‐sitosterol, diosgenin, formononetin, kaempferol, luteolin and quercetin—were chosen for molecular docking verification with the key targets AKT1, CAV1, DPP4, IL6, INSR, MAPK8, MMP9, PPARG and SLC2A4, which are considered potential targets in the treatment of diabetes. The binding energy (Vina score) calculated by AutoDock Vina was used to estimate the bonding activity between the docking molecules, with a smaller Vina score indicating stronger bonding activity as well as a higher affinity and more stable structure between the ligand and receptor. Compared to the reference drug, these important compounds showed a stronger affinity for the key targets (<−5.0 kcal/mol, Table 5), which demonstrated good docking and high affinity. The top three Vina scores for each important compound and their selected targets are visualized in Figure 6, which indicates that the docking point and structure of small‐molecule ligands and protein receptors were stable.

**TABLE 5:** Target molecule docking results for candidate core active constituents.

Compound name	Affinity (kcal /mol)									
	AKT1	CAV1	DPP4	IL6	INSR	MAPK8	MMP9	PPARG	SLC2A4	Mean
β‐Sitosterol	−7.3	−5.0	−6.4	−5.0	−6.7	−5.2	−5.2	−7.7	−6.6	−6.1
Diosgenin	−9.7	−7.8	−9.0	−7.0	−9.5	−10.4	−8.4	−8.9	−8.4	−8.8
Formononetin	−8.1	−6.7	−8.2	−6.3	−7.5	−6.5	−7.6	−6.8	−8.3	−7.3
Kaempferol	−7.8	−6.3	−7.8	−6.6	−7.7	−8.4	−8.0	−6.9	−8.9	−7.6
Luteolin	−8.0	−6.0	−8.7	−7.7	−7.9	−7.7	−8.1	−7.2	−8.6	−7.8
Quercetin	−8.2	−5.9	−7.8	−6.9	−7.4	−8.5	−8.1	−7.1	−8.6	−7.6
Mean	−7.6	−5.8	−7.5	−6.2	−7.2	−7.2	−7.1	−7.0	−7.6	
Metformin	−5.1	−3.7	−5.0	−4.8	−5.1	−4.7	−5.3	−5.0	−4.7	−4.8

ADMET analysis is an important tool in drug discovery. The SwissADME database was used for ADMET analysis, and the results revealed that the important compounds have excellent pharmacokinetic properties.^29^ As we can see in Table 6, all six selected drug candidates showed no side effects in terms of their pharmacokinetic properties in the various prediction models, including P‐glycoprotein substrates, blood–brain barrier penetration, gastrointestinal absorption and human oral absorption. Furthermore, all the important active constituents showed good biocompatibility in the various toxicity prediction models, and none of them showed toxic behaviour such as reverse mutation, hepatotoxicity and cytotoxicity. Formononetin, luteolin and quercetin shows ‘active’ in carcinogens and mutagenicity predictions. According to The toxicity radar chart, it is not a strong confidence of positive toxicity results. Several researches have proved formononetin, luteolin and quercetin were high biocompatibility.^30^, ^31^, ^32^

**TABLE 6:** ADMET profiling of compounds.

Compounds	β‐Sitosterol	Diosgenin	Formononetin	Kaempferol	Luteolin	Quercetin
GI absorption	High	High	High	High	High	High
BBB permeant	No	Yes	Yes	No	No	No
P‐gp substrate	No	No	No	No	No	No
Human oral abs	High	High	High	Media	Media	Media
CYP1A2 inhibitor	No	No	Yes	Yes	Yes	Yes
CYP2C19 inhibitor	No	No	No	No	No	No
CYP2D6 inhibitor	No	No	Yes	Yes	Yes	Yes
CYP2C9 inhibitor	No	No	No	No	No	No
Predicted LD_50_	890 mg/kg	8000 mg/kg	2500 mg/kg	3919 mg/kg	3919 mg/kg	159 mg/kg
*Toxicity*						
Reverse mutation Ames test	Non‐Toxic	Non‐Toxic	Non‐Toxic	Non‐Toxic	Non‐Toxic	Non‐Toxic
Hepatotoxicity	Inactive	Inactive	Inactive	Inactive	Inactive	Inactive
Carcinogens	Inactive	Inactive	Active(0.50)	Inactive	Active(0.69)	Active(0.69)
Mutagenicity	Inactive	Inactive	Inactive	Inactive	Active(0.52)	Active(0.52)
Cytotoxicity	Inactive	Inactive	Inactive	Inactive	Inactive	Inactive

Abbreviations: BBB, blood–brain barrier; GI, gastrointestinal; Oral abs, oral absorption.

### 3.8 Molecular simulations and binding free energy calculations

**FIGURE 7:**
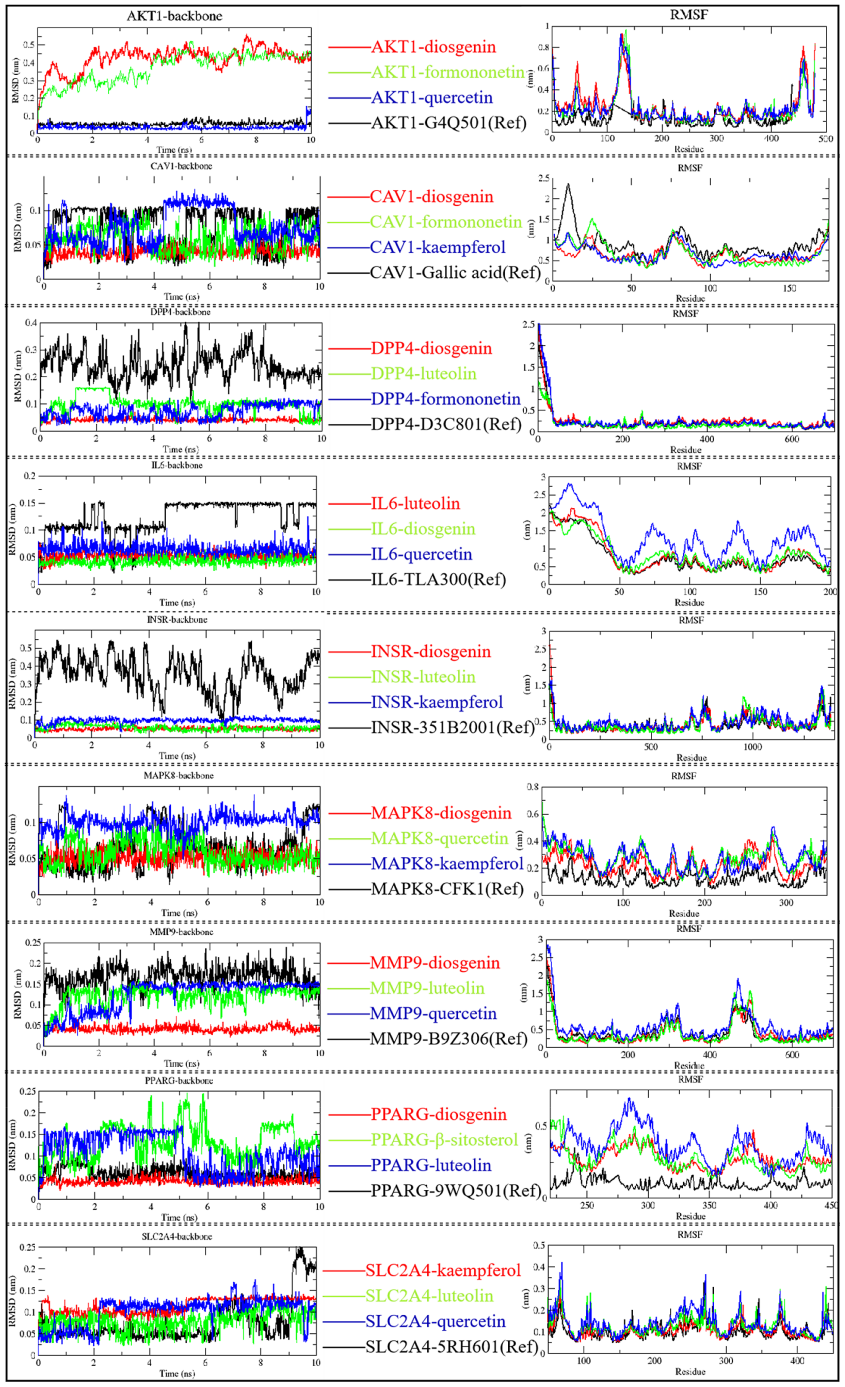
RMSD and RMSF plot of selected receptor‐ligand complexes during molecular dynamics simulations. (Ref): reference system.

According to molecular docking results, the top three protein‐ligand complexes in each group, ranking by docking affinity, were selected for MD simulations. The stability of the system was tested by calculating the root mean square deviation (RMSD) in 10 ns of the simulation trajectory. Root mean square fluctuation (RMSF) were used to analyse the fluctuation and flexibility of each complex during simulation. As show in Figure 7, the performances of each receptor‐ligand complex was independent. However, in each group, we can find out more than one protein‐ligand complexes which were more stable than reference system. These complexes consistently maintain a slighter fluctuation range during the simulations (RMSD <0.1 nm). Among them, AKT1‐quercetin, CAV1‐diosgenin, DPP4‐diosgenin, IL6‐luteolin, INSR‐diosgenin, MAPK8‐diosgenin, MMP9‐diosgenin, PPARG‐diosgenin and SLC2A4‐luteolin were marked as representative complexes. RMSF results indicate that representative complexes appear nearly the same peak and valley values as the reference system. It means the effect site of those compounds to target receptor are similar to referenced inhibitor. They may have similar mechanism of pharmacodynamics to references.

Meanwhile, the binding energies of these complexes were calculated using the GMX‐MM/PBSA method. From Table 7, AKT1‐quercetin, CAV1‐diosgenin, DPP4‐diosgenin, IL6‐luteolin, INSR‐diosgenin, MAPK8‐diosgenin, MMP9‐diosgenin, PPARG‐diosgenin and SLC2A4‐luteolin exhibit lower binding free energy comparing to members in the same group. This phenomenon indicates that these compounds have a strong binding affinity to the target receptor. Besides, the contribution of this binding affinity is mainly derived from van der Waals force and electrostatic interactions. Therefore, based on the collected results, diosgenin, quercetin and luteolin were further screened as the core components of fenugreek in anti‐diabetes. These core components can bind with key targets to form stable complexes, which in turn exert antidiabetic effects.

**TABLE 7:** The binding free energy of receptor‐ligand complex system by GMX‐MM/PBSA method.

Energy components (Kcal/mol)						
Target	Compounds	△E_vdw_	△E_elec_	△G_gas_	△G_solv_	△G_bind_
AKT1	Diosgenin	−17.13	−1.6	−18.73	12.84	−5.90
	Quercetin	−21.65	−55.74	−77.39	69.85	−7.54
	Formononetin	−29.70	−15.10	−44.80	35.38	−9.43
	G4Q501 (Ref)	−74.08	−30.65	−104.74	72.42	−32.32
CAV1	Diosgenin	−18.84	−0.77	−19.61	3.78	−15.83
	Formononetin	−20.98	−21.93	−42.91	29.22	−13.69
	Kaempferol	−23.08	−13.39	−36.47	25.61	−10.68
	Gallic acid (Ref)	−4.6	−35.76	−40.39	36.87	−3.52
DPP4	Diosgenin	−22.39	−4.23	−26.62	13.87	−12.75
	Luteolin	−22.37	−41.20	−63.57	48.42	−15.15
	Formononetin	−29.58	−12.41	−41.99	29.98	−12.01
	D3C801 (Ref)	−11.13	−5.98	−17.11	12.14	−4.97
IL6	Luteolin	−28.09	−63.02	−91.11	70.83	−20.28
	Diosgenin	−15.82	−0.82	−16.64	10.54	−6.10
	Quercetin	−21.93	−16.47	−38.40	32.91	5.49
	TLA300 (Ref)	−0.34	−0.46	−0.80	0.75	−0.05
INSR	Diosgenin	−30.51	−9.38	−39.89	32.01	−7.89
	Luteolin	−26.59	−28.42	−55.01	52.75	−2.26
	Kaempferol	−32.17	−15.76	−47.93	48.85	0.92
	351B2001 (Ref)	−6.26	−3.62	−9.88	5.44	−4.44
MAPK8	Diosgenin	−29.50	−8.24	−37.74	15.06	−22.68
	Quercetin	−28.18	−22.50	−50.68	40.32	−10.45
	Kaempferol	−31.10	−23.73	−54.83	47.00	−7.83
	CFK1 (Ref)	−30.66	−24.61	−55.27	30.39	−24.89
MMP9	Diosgenin	−35.70	−19.53	−55.23	33.65	−21.58
	Luteolin	−30.70	−18.97	−49.67	42.49	−7.19
	Quercetin	−32.78	−35.69	−68.47	59.17	−9.30
	B9Z306 (Ref)	−0.29	−0.53	−0.81	0.66	−0.15
PPARG	Diosgenin	−15.13	−1.60	−16.73	12.34	−4.40
	β‐sitosterol	−24.42	−0.08	−24.50	6.63	−17.87
	Luteolin	−21.66	−19.28	−40.94	33.49	−7.45
	9WQ501 (Ref)	−28.16	−10.92	−32.11	19.18	−12.93
SCL2A4	Kaempferol	−32.56	−8.52	−41.08	36.97	−4.11
	Luteolin	−32.13	−12.42	−44.55	37.83	−6.72
	Quercetin	−28.84	−8.57	−37.42	30.11	−7.30
	5RH601 (Ref)	−45.60	−20.93	−66.53	57.47	−9.06

Abbreviations: ∆E_vdW_, van der Waals energy; ∆E_elec_, electrostatic energy; ∆G_gas_, gas‐phase free energy; ∆G_solv_, solvation free energy; ∆G_bind_, total binding free energy; (Ref), reference system.

### 3.9 Effect of fenugreek on glucose consumption in IR HepG2 Cells

**FIGURE 8:**
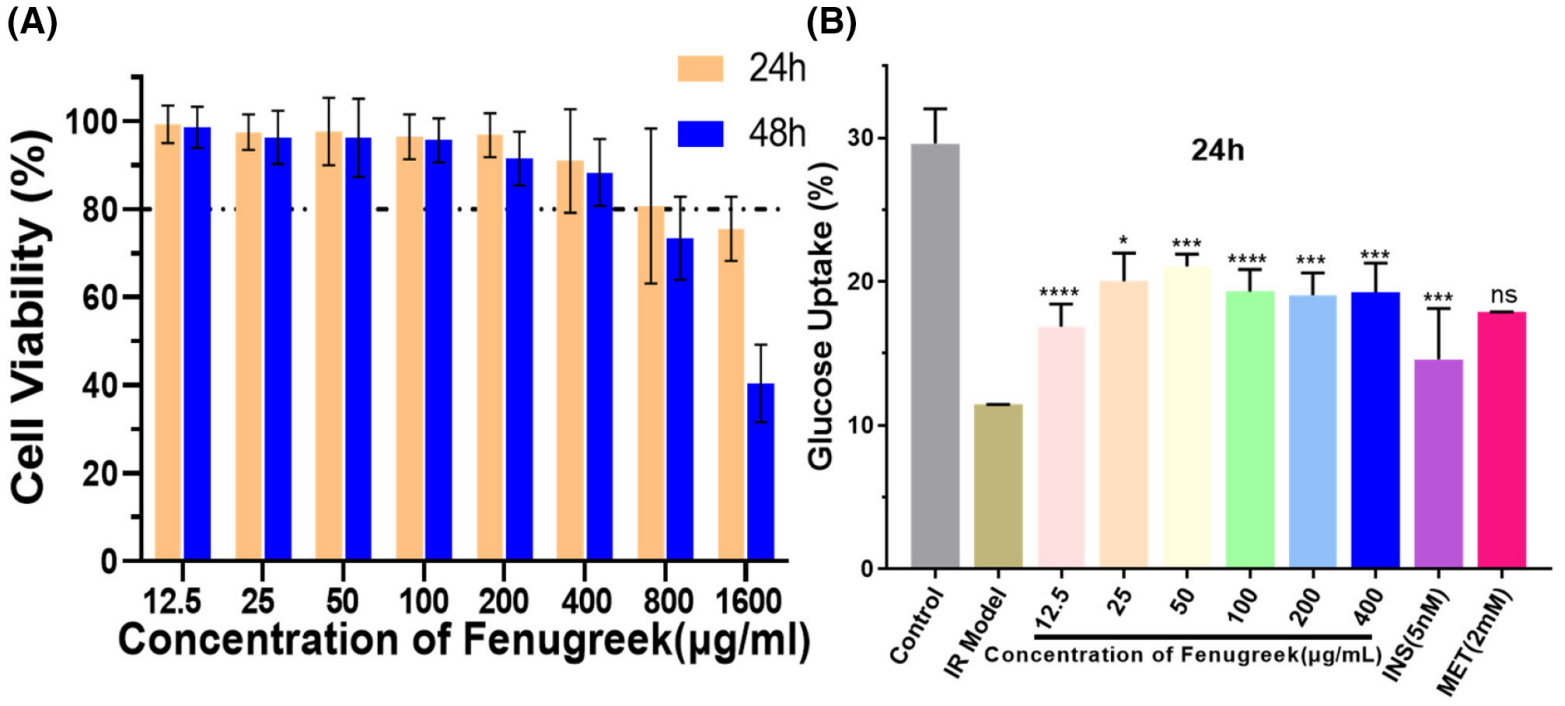
Effects of fenugreek on cell viability and glucose uptake in IR‐HepG2 cells. (A) Cell viability of HepG2 cells cultured in different concentrations of fenugreek from 0 to 1600 μg/mL for 24 h and 48 h. (B) Glucose consumption of IR HepG2 cells incubated with or without fenugreek for 24 h (****p < 0.001 vs. the model group, ***p < 0.01 vs. the model group, *p < 0.05 vs. the model group).

Figure 8A illustrated that, the viability of HepG2 cells decreased with increasing concentration of fenugreek. Comparing with normal cells, significant cytotoxicity appeared at concentrations upper than 400 μg/mL of fenugreek. It suggests that the safety treatment dosage of fenugreek for HepG2 cells was lower than 400 μg/mL. As shown in Figure 8B, by comparing to IR model, the glucose consumption was significantly increased in all of the fenugreek treated group. Thus, fenugreek may contribute to improve the glucose uptake in IR‐HepG2 cells.

## 4 DISCUSSION

Previous research has investigated the anti‐diabetic effects of fenugreek.^11^ In addition, extractions derived from fenugreek have been used for the treatment of diabetes, with good results. The therapeutic effects of fenugreek manifest mainly as improvements to insulin sensitivity and resistance as well as reduced fasting blood glucose and HbA1c levels.^33^ These pharmacodynamic activities may be related to the flavonoids, alkaloids, volatile oils and unsaturated fatty acids derived from fenugreek.^14^ However, which compositions are most effective, the potential target genes and the mechanisms of action are not fully understood, which limits the application of fenugreek.

In recent years, molecular docking technology and network pharmacology have been used to screen for active constituents and potential targets of action, to evaluate the degree of compound–target association, and to explain the pharmacological mechanisms of action in traditional Chinese medicines.^34^ These tools are helpful for accelerating the clarification of the pharmacological mechanisms in traditional Chinese herbal medicines and the development of new drugs. The findings of this study can serve as a touchstone for the preliminary screening of anti‐diabetic active compounds in fenugreek as well as provide a new therapeutic concept for further exploring the anti‐diabetic mechanisms of action in fenugreek.

This study systematically investigated the pharmacological mechanism of fenugreek in the treatment of diabetes. First, the active constituents were obtained from the TCMSP, CNKI and PubMed databases. Then, the physical and chemical information of these compounds was analysed, and 19 compounds—mairin, β‐sitosterol, irolone, formononetin, calycosin, kaempferol, diosgenin, gallic acid‐3‐O‐(6’‐O‐galloyl)‐glucoside, luteolin, hiyodori lactone A, tigogenone, isovitexin, neotigogenin, quercetin, vicenin‐2, vitexin‐7‐glucoside_qt, isoorientin, vitexin and 4‐hydroxyisoleucine—were determined to be active constituents, which is consistent with the results of a recent study.^35^ Among these active constituents, 4‐hydroxyisoleucine, quercetin and diosgenin have been shown to have anti‐diabetic activity and may exert additional anti‐diabetic effects by affecting key genes such as AKT1, DPP4, INSR, MAPK8, MMP9, PPARG and SLC2A4. Furthermore, the results of network pharmacological analysis revealed that these compounds exert anti‐diabetic effects by modulating the role of target proteins in various metabolic pathways. Meanwhile, the Compound–Gene–Pathway network suggested that six important active constituents—β‐sitosterol, diosgenin, formononetin, kaempferol, luteolin and quercetin—are included in the candidate active constituents responsible for the anti‐diabetic effects of fenugreek. Moreover, the ADMET analysis of active constituents exhibited properties such as intestinal absorption, oral bioavailability, P‐glycoprotein substrate and blood–brain barrier penetration. More importantly, based on cytotoxicity model prediction analysis, the six important active constituents showed a high LD_50_ (>150 mg/kg) and no cytotoxicity. In addition, molecular docking and dynamics simulation further screen out that diosgenin, quercetin and luteolin show stronger binding capacity with the corresponding targets. These results suggest that the connection between the selected core active constituents and the core diabetes‐related target genes is stable, indicating that the core active compounds in fenugreek can bind to the key genes involved in diabetes mellitus and induce anti‐diabetic activity. Futhermore, cell culture results demonstrated that fenugreek not only has good biological safety in vitro, but also can effectively improve the glucose consumption of insulin resistance HepG2 cells. Thus, our results further suggest that these core active constituents might be candidate drugs for the treatment of diabetes and its complications.

According to the GO functional enrichment analysis, the anti‐diabetic GO activities of fenugreek affect mainly BP, including response to peptides, response to oxygen levels, cellular response to chemical stress, positive regulation of protein phosphorylation, regulation of the apoptotic signalling pathway and regulation of the circulatory system. Based on the results of KEGG pathway enrichment analysis, in addition to the AGE‐RAGE pathway, which is closely related to diabetes, other related signalling pathways were enriched in terms of diabetes complications, including the cancer signalling pathway, the FoxO signalling pathway, the HIF‐1 signalling pathway, the NF‐κB signalling pathway and lipids and atherosclerosis. A growing number of studies has shown that the AGE‐RAGE signalling pathway plays a key role in the generation of vascular complications related to diabetes, including atherosclerosis and kidney disease.^36^

In terms of increasing the levels glucose and other reducing sugars, AGE‐RAGE signalling is strongly amplified, thereby inducing the release of NAD(P)H oxidase and increasing the production of reactive oxygen species. This condition may cause severe oxidative stress as well as vascular endothelial and peripheral nerve dysfunction, which in turn can lead to serious diabetic complications, including diabetic foot, diabetic nephropathy and diabetic myocardial injury.^37^ Additionally, numerous reports have suggested that there is an amplified synergy between the AGE‐RAGE signalling pathway and the TNF‐α signalling pathway. Increased TNF‐α levels impair insulin signalling through serine phosphorylation, which induces insulin resistance and leads to the development of T2DM.^38^ During the development of diabetes, the nuclear factor is closely associated with inflammation and oxidative stress activation and plays a key role in the expression of cytokines such as TNF‐α, IL‐6 and IL‐1B as well as adhesion molecules such as ICAM1.^39^ Zheng et al.^40^ reported that the nucleus transfer ratio of the endothelial cell protein NF‐κBp65 was reduced under high‐glucose conditions by pre‐treatment with NF‐κB inhibitors. Subsequently, vascular endothelial damage was reduced. The American Diabetes Association and the American Cancer Society have suggested that T2DM and cancer have many overlapping pathogenic factors.^41^ Indeed, when patients are treated for some of the most severe types of cancer, they are three times more likely than other patients to develop diabetes. Last but not least, diabetes is a chronic inflammatory disease. In the present study, molecular docking technology and network pharmacology were used to reveal the mechanism by which fenugreek exerts its anti‐diabetic effects. The selected key target genes, biological processes and signalling pathways are closely related to inflammation. Therefore, we hypothesize that the anti‐diabetic effect of fenugreek was exerted through the inhibition of inflammatory signalling pathways and by regulating the effects of inflammatory factors on pancreatic tissues and fat. However, this hypothesis requires subsequent experimental verification.

## 5 CONCLUSION

We used molecular docking, molecular dynamics simulation and network pharmacology to conduct a preliminary study of the active constituents and mechanisms of action of fenugreek in the treatment of diabetes. A total of 19 active constituents were selected, three of which—diosgenin, luteolin and quercetin—were screened as core active constituents. In addition, a total of 71 common target genes were screened, of which ESR1, CAV1, VEGFA, TP53, CAT, AKT1, IL6 and IL1 were predicted to be the core target genes. Furthermore, KEGG pathway enrichment analysis showed that fenugreek exerted anti‐diabetic effects by inhibiting inflammatory signalling pathway, reducing inflammatory factor expression and protecting the vascular endothelium, peripheral nerves and islet cells against inflammatory cytokines. The signalling pathways include the AGE‐RAGE signalling pathway, the FoxO signalling pathway, the HIF‐1 signalling pathway, the NF‐κB signalling pathway and lipids and atherosclerosis. Overall, this study provides profound insights into the biological activity, potential targets of interaction, and mode of action of fenugreek against diabetes. It also provides a theoretical basis for exploring the role and related mechanisms of fenugreek in the treatment of diabetes. Moreover, those selected constituents are biocompatible and antidiabetic. Thus, they could be potential therapeutic agents for diabetes mellitus. Our results may help for developing anti‐diabetic dietary supplements or drugs base on fenugreek. Going forward, we will conduct animal and clinical studies to validate the screening targets based on this predictive analysis, with the aim of providing additional scientific evidence for the clinical application of fenugreek in the treatment of diabetes.

## AUTHOR CONTRIBUTIONS

**Wenfeng Luo:** Conceptualization (lead); data curation (equal); project administration (equal); resources (equal); writing – original draft (equal). **Jie Deng:** Data curation (equal); formal analysis (equal); funding acquisition (equal); software (equal). **Jiecheng He:** Data curation (equal); methodology (equal); software (equal); visualization (equal). **Liang Yin:** Data curation (equal); investigation (equal); visualization (equal). **Rong You:** Conceptualization (equal); methodology (equal); supervision (equal); writing – review and editing (equal). **Lingkun Zhang:** Investigation (equal); software (equal); validation (equal). **Jian Shen:** Resources (equal); software (equal); validation (equal). **Zeping Han:** Conceptualization (equal); investigation (equal); software (equal). **Fangmei Xie:** Data curation (equal); resources (equal). **Jin‐Hua He:** Funding acquisition (equal); visualization (equal); writing – review and editing (equal). **Yanqing Guan:** Conceptualization (equal); funding acquisition (equal); project administration (equal); writing – review and editing (equal).

## FUNDING INFORMATION

This work was supported by the Science and Technology Program of Panyu (2019‐Z04‐07, 2019‐Z04‐35, 2019‐Z04‐84, 2020‐Z04‐054), the Science and Technology Project of the Guangzhou Health Commission (No. 20211A011114), the Science and Technology Program of Guangzhou (No. 202002020023), the General University Youth Innovative Talent Project of Guangdong Province (No. 2022KQNCX281).

## CONFLICT OF INTEREST STATEMENT

The authors have no conflicts of interest to declare.

## Data Availability

Data available on request from authors.
